# ‘They Take the Mum Off You When You Come In’: An Ethnographic Study of Parent Experiences of Medicines Safety Systems in English Hospitals

**DOI:** 10.1111/hex.70161

**Published:** 2025-02-09

**Authors:** Adam Sutherland, Denham L. Phipps, Stephen Tomlin, Suzanne Grant, Joanne Hughes, Joanna Chambers, Susan Kafka, Heidi Ridgewell, Darren M. Ashcroft

**Affiliations:** ^1^ NIHR Greater Manchester Patient Safety Research Collaboration Manchester UK; ^2^ Division of Pharmacy and Optometry, School of Health Sciences, Faculty of Biology, Medicine & Health University of Manchester Manchester UK; ^3^ Pharmacy Department, Royal Manchester Children's Hospital Manchester University NHS Foundation Trust Manchester UK; ^4^ Medicines Optimisation Research Group, School of Pharmacy & Medical Sciences, Faculty of Life Sciences University of Bradford Bradford UK; ^5^ Great Ormond Street Children's Hospital NHS Foundation Trust London UK; ^6^ Children's Medicines Research & Innovation Centre London UK; ^7^ Division of Population Health and Genomics, School of Medicine University of Dundee Dundee UK; ^8^ Mother's Instinct Cambridge UK; ^9^ For the MOPPEt Family Forum, Division of Pharmacy and Optometry, School of Health Sciences, Faculty of Biology, Medicine & Health University of Manchester Manchester UK

**Keywords:** children, ethnography, medication safety, parents, self‐administration of medicines

## Abstract

**Introduction:**

Medication safety in healthcare settings is a persistent problem, and children may be at greater risk of harm than adults. Most existing research examining medication safety for hospitalised children is from the perspective of healthcare professionals and organisations. This study aimed to ethnographically explore parent and staff perspectives on the role of parents in medication safety in the paediatric hospital setting.

**Methods:**

230 h of ethnographic observation and 19 semi‐structured interviews with clinical staff and parents were conducted over paediatric wards in three acute hospitals in Northern England between October 2020 and May 2022. Data was organised and coded using NVivo and analysed thematically.

**Results:**

Three main themes were identified: (1) Capacity and Capability: Parents were often assumed to be incompetent by organisational policies and managers but at the same time were co‐opted to undertake medication processes to meet operational needs. Parental experience was often ignored or judged negatively. When things went wrong parents were sometimes blamed. (2) Communication: parents were seldom meaningfully involved in decisions about their children's medication or provided with appropriate information unless requested. Parental medication histories were treated with suspicion and validated against inaccurate records. (3) Agency and Autonomy: parents often wanted to participate in their child's care but were expected to be passive observers.

**Conclusions:**

Medication safety for children is a social phenomenon involving healthcare professionals and parents. However, parents are often relegated to a passive role by healthcare staff. We posit that this represents an example of epistemic injustice in the way parents are assumed to be incompetent outsiders with no understanding of the medical care of their children, despite them offering resilience for medicines safety. We recommend further exploration of how parents contribute to resilience and safety for children in hospital and the barriers to this, and how health services can safely support increased engagement and involvement of parents in the care of their children while in hospital.

**Patient or Public Contribution:**

Parents contributed to the analysis and interpretation of the data collection and have contributed to the preparation of the manuscript.

## 1 Introduction

Medication safety is a continuing challenge for healthcare systems. It is estimated that 6% of patients suffer severe avoidable harm during their care, and 25% of these events are related to medication [1]. Harm is common among hospitalised children, and is estimated to currently affect between one and two per cent of patients [2]. Additionally, the incidence of potentially avoidable medication‐associated harm (i.e., medication errors that can be intercepted and prevented) among children and young people would appear to be higher than in other populations [3]. There are systemic issues associated with medicines safety for children and young people that may not be relevant to other healthcare service users. For instance, while there is a continued requirement for bespoke weight‐based medication dosing for children, there is still a relative lack of evidence‐based guidelines for medicines use in children and young people, and many children in hospital are pre‐verbal and cannot support medication administration themselves [4, 5]. The reliance on unlicensed and off‐label medicines in this population contributes to relatively high rates of adverse drug reactions (ADRs), 25% of which are severe enough to warrant medical intervention [6]. These patient‐centric issues are further compounded by the structure of care provided to children, with care provided from multiple institutions which requires navigation and negotiation by families and caregivers alike [7].

The perspective and experience of patients and families have been a topic of interest in patient safety research for over 20 years [8, 9]. Service users have been recognised as providing an important alternative perspective on patient safety, and can identify patient safety incidents that would otherwise go unnoticed [10]. Further, patients also have differing experiences of the contributory factors to medication safety events than those perceived by healthcare services [11]. However, these studies have focussed on adult patients. There are strong arguments that robust and deep analysis and understanding of patient and service user experiences improve patient safety [12, 13, 14]. There have been several studies exploring this phenomenon, where patients contributed purposefully to medicines safety through communication and trust with their healthcare professionals, but have also identified how patient perceptions of safety are poorly accommodated within healthcare systems [15, 16, 17].

Attempts to operationalise patient participation in safety have often been described as ‘knee‐jerk reactions to adverse events’ [18]. More specific to paediatric practice, Stebbing (2007) has advocated for the involvement of parents in paediatric medication safety but only in the context of them voicing concerns about medication dosing [19]. This approach to patient and carer involvement has been justified by concerns about the tokenisation of their involvement, and that families may report safety events that are unimportant or be subjected to burdens that they may not be able to bear [20, 21, 22]. These arguments raise a legitimate concern that constructions of ‘harm’ and ‘safety’ exclude non‐clinical harms that patients may view as significant, but which wider systems do not [17].

Many studies of clinical risk and safety have been framed within a ‘biomedical model’ focusing only on physical harms that can be seen and measured and from the perspectives of wider systems and clinicians [23, 24]. Importantly, this model assumes that outcomes are simple cause‐and‐effect relationships [25]. However, it has been argued more recently that patient safety is a collaborative process and practice that is achieved collectively within the context of complex socio‐technical systems. Of increasing importance in the study of patient safety is the concept of ‘resilience’ – that safety is an emergent property of a complex system, and that operators within the system can and do make in situ changes in their practice to prevent adverse outcomes [26, 27]. As such, it is advantageous to include patients and carers in safety research to provide a rich and deep understanding of ‘safety’ within complex healthcare settings. [28]

The aim of this paper is to explore the experiences, perceptions and practices of medicines safety for hospitalised children and young people from the perspective of parents and carers and the staff working with them, and to describe the contribution of this participant group to medicines safety, using parents as analysts of this data.

## 2 Methods

The study reported in this is drawn from a larger ethnographic study exploring medicines safety in English paediatric hospitals, which has been described further elsewhere. The study was approved by the Yorkshire and Humber Leeds West Research Ethics Committee.

### 2.1 Data Collection

The study was conducted between October 2020 and May 2022 in three hospitals across the north of England. Study sites were selected using a maximal variation purposeful sampling strategy based on the following characteristics:

- Diversity of organisational characteristics to include a standalone tertiary children's hospital (CH1), a tertiary children's hospital as part of a larger multi‐speciality hospital group (CH2) and a small general hospital (GH1).
- Population diversity across these settings (economic, cultural and social).
- Geographical relationship with children's hospitals selected to minimise cultural mixing, and the general hospital selected to reflect referral patterns into a participating tertiary hospital, and the effect of cultural mixing.

The detailed characteristics of the included sites are summarised in Table 1.

**Table 1:** Characteristics, location and size of study sites.

	Location	Hospital	Unit size
GH1	A small town in England; pop. 55,000	District general (245 beds). Neonatal Unit closed prior to initiation. Low technology medicines management systems in place – paper prescribing, conventional medicines storage cupboards under lock and key.	12 beds, and a six‐bed assessment area
CH1	A post‐industrial city of England; pop. 500,000	Standalone tertiary children's (270 beds). Neonatal care provided off‐site. High‐technology medicines management systems in place – electronic prescribing, automated dispensing cabinets, conventional medicines storage cabinets behind proximity lock systems, barcode medicines administration.	28 beds
CH2	Medium‐sized city England; pop. 800,000	The children's hospital (286 beds) on a city centre hospital site (1100 beds); part of a multi‐hospital trust (2500 beds). Medium technology medicines management systems in place – electronic prescribing, conventional medicines storage cupboards under lock and key.	12 beds. Other secondary care admissions were distributed elsewhere in specialist areas based on bed availability.

A focused ethnographic methodological approach was adopted that used targeted observations to provide deep and focused data pertinent to the problem [29, 30, 31]. This involved the following:

- Relatively short observation periods (< 90 h in each site),
- A single observer with experience in the field and
- A clearly defined problem – in this case, how the work of medicines safety is undertaken in different sites, and by whom.

Non‐participant observations were carried out by the lead researcher to explore how the work of medicines safety was done in practice. Informal ethnographic interviews were used during these observations to clarify with participants what was being observed [32]. Semi‐structured interviews were additionally used to provide depth on the observations and in particular to explore the perceptions and experiences of medicines safety described by the participants.

With permission, 404 participants (medical, nursing and pharmacy staff, support staff, and families) were observed by the lead researcher. These observations explored all the work around medicines – prescribing, preparation, administration and overall monitoring of medicines use – and involved all those who undertook this work. Observation participants gave verbal consent. We conducted a number of ethnographic conversations with a wide range of participants including 10 parents of children in hospital (nine mothers, one father). These were informal conversations following the description of Spradley [32] that formed part of the observations and were recorded as part of the fieldnotes. In addition, 19 participants including healthcare professionals (e.g. nurses, doctors, pharmacists) and parents took part in formal semi‐structured interviews (Table 2). These participants were identified and recruited through the observations, and in the case of parents through links with community groups and support networks.

**Table 2:** Purposive sample and characteristics of interview participants.

Designation	Number (%)
Nurse	5 (26%)
Ward	3
Managerial (e.g. MSO)	2
Doctor	5 (26%)
Junior	2
Consultant	3
Pharmacist	5 (26%)
Parent	3 (16%)

Observation data and the content of ethnographic conversations were initially captured by the lead researcher as fieldnotes using a secure tablet computer. Separately a reflexive diary of questions was maintained to guide future observations and to ensure rigour in the ongoing data collection and analysis. The fieldnotes taken on the tablet computer and these reflections were later typed up as full fieldnotes for analysis. Interviews were conducted in person (for staff) or virtually via Zoom (for parents) and audio‐recorded with permission. They were then transcribed verbatim for analysis.

### 2.2 Data Analysis

Qualitative data was organised and managed using NVivo version 12 [33]. Analysis was conducted inductively using Braun and Clarke's Thematic Analysis approach [34]. Data was initially coded by a single analyst using an inductive coding approach to create an initial codebook. The codebook was reviewed and agreed across all analysts and was then organised into analytical themes and categories. To support this process, two analytical teams were convened. The first consisted of the lead author and methodological experts – a social anthropologist, a professor of pharmacy, a chartered psychologist and ergonomist and a patient safety advocate with lived experience of paediatric medication‐related harm. This analyst received specialist training in qualitative data analysis.

A second analytical team made up entirely of parents with experience of children's hospital care and medicines was also convened. This ‘Family Forum’ was recruited from interested observation participants and local clinical or support networks (Table 3). The Family Forum met during the spring of 2022.

**Table 3:** Composition of the family forum.

Parent	Occupation	Location	Relevance to the study
Mother of a fit and well child	Healthcare professional	Scotland	Historical experience of cardiac surgery in CH1
Mother of a child with medical complexity	Charity case worker	Yorkshire and Humber	Previous in‐patient spells in CH2
Mother of a child with medical complexity	Full‐time carer	Yorkshire and Humber	On‐going in‐patient spell in CH2
Mother of a child who passed away because of medication‐related harm.	Patient Safety Advocate	East of England	Lived experience and lay researcher

Excerpts from the data that related to parents' experience and activity in the ward environment were provided in advance of sessions, and forum members were asked to read them and consider their understanding of the circumstances and potential meaning of the events from the parent's perspective. They were provided with a copy of the codebook and were asked to consider how those codes reflected their interpretations of the data. They were encouraged to suggest their own codes and descriptions which were then discussed among the forum members and the codebook was adjusted and agreed by consensus. These parental perspectives and insights were acknowledged by the academic analytical group during later data sessions and were incorporated into the analysis. Participant validation was provided using two Experience Based Co‐Production workshops held in the spring of 2023 which involved all members of the family forum and invited healthcare professionals.

## 3 Results

Parents identified and described three aspects of their experience in medicines safety – Capacity and Capability, Agency, and Autonomy and Communication.

### 3.1 Capacity and Capability

At an organisational level, concerns for patient safety were demonstrated through a focus on accountability and documentation. There was no consistent approach to the definition and description of parent administered medicines in any research site. One took a pragmatic view asking parents to sign a ‘waiver’ taking responsibility for medicines administration, while another demanded assessment of parental mental capacity and capability. One nurse described how the assessment of parent capacity and capability for administration of medicines was conducted in their ward.

I ask one of the nurses about the self‐med policy and checklist. ‘It's really bloody patronising. There's really very little in there about medicines and doses and stuff, and more about “Are you suicidal today…” It just feels really weird asking those questions of parents who are so on‐board with their child's medicines’.

(Fieldnote observation, Nurse, CH1)

There was a suggestion in the observations that different wards within a single site had a different approach to self‐medication.

…Yeah so we're not normally looked after on [this ward] we get seen on [another ward] and there, I think because they know us they're happy to just let us, y'know, crack on. We sign a waiver, but then we came down here and they were, like, ‘Oh yeah, we don't do that here…it's just not very consistent’.

(Interview 17, Parent)

Some managers viewed parent administration as inequitable and creating opportunities for error and confusion. They were also concerned that the patient‐centred care and parental self‐administration contributed to unequal treatment among patients with families of well‐known children getting different treatment to those families who are in hospital for a single episode.

I do get the feeling that we are catering to the needs of some parents at the expense of others…

(Fieldnote discussion, Nurse Manager, CH2)

Particularly those families of children with medical complexity, or chronic illnesses who required frequent hospital visits, they developed relationships with the staff on the ward as a result of their experiences and learned to speak to clinical staff assertively using clinical terms to advocate for their child's safety.

Over the years I've got more experience… and yeah, I found my voice… because [in the past] he's had adverse drug reactions which they just put down to autonomic seizures, but he was toxic off his carbamazepine. I wasn't impressed. I googled it and found that there was a blood test and no one did it. (Interview 15. Parent)

Spoke to a parent with her child admitted overnight. They have a heart condition and mum remarks that they are on medicines for it.

Yeah, I always give the medicines, but I'll just tell the nurses when I've done it, and they're okay with that.

(Fieldnote discussion, Parent, CH1)

During the course of the study, it became apparent that while parents were involved in medication processes in hospital, it was also the case that when there was an error or event, the parents would be ‘blamed’ by the clinicians around them. Yet it was clear that these ‘events’ were mostly related to the way clinicians communicated with parents, and the way that they were incorporated into the care environment.

We've changed that dose, that medicine… have we communicated that change to the parent? What is their level of understanding of that change…? How do we record that?

(Interview 11, Medicines Safety Officer)

It was very clear that in all sites the mechanisms for communication with parents and families were limited to ward rounds and opportunistic interactions with care professionals when they were available. Again, assumptions were made about the understanding of roles and responsibilities. In a situation with a child admitted with gastroenteritis, the nursing staff had decided that the parent should administer the oral glucose solution to their child overnight. However, this did not happen, and the child became hypoglycaemic.

…When questioned by the consultant it emerged that nursing staff had told the parent to administer the appropriate oral fluids, but they had fallen asleep “…because she hasn't slept in 3 days…”

(Fieldnote observation, GH1)

This raises an interesting reflection on task allocation and risk management. In all sites, managers and senior clinicians were concerned about parent administration of medicines because fundamentally they could not regulate it. This came down to an inability to ensure adequate and unambiguous communication with parents.

It was also observed that parents would often intervene to keep their children safe either by alerting staff to medication that had been omitted or to identify documentation errors but there was evidence again that these exhortations from parents were ignored or overlooked. There were reports from some parents and from staff that these warnings were overlooked because staff distrusted parents or assumed an emotional sensitivity on the part of the parent that tainted their interpretation of parent advocacy.

[I felt] awful… like I didn't have any control because no one was listening to me and I just felt like [all I could do] was shout and scream until they listened.

(Interview 19, Parent)

I always tell my nurses to use their parents… some of them are very well informed and they'll move stuff around you but… some people feel that they're being policed by the family…

(Interview 11, Medicines Safety Officer)

Yet parents argued that without them, medications would be administered late or incorrectly, or not at all. Their prior experience of hospital care and the needs of their child empowered them to speak up and challenge practice that they were concerned about and their previous experience supported them in finding their voice.

If I leave it to the nurses everything will be late… 60% within an hour but the rest… A medicine was once missed and we found it in a syringe in the sheets four hours later… (Fieldnote discussion, Parent, CH2)

I: So how did you “find your voice”

P: I think it was because I knew in my gut that things weren't right, and I just had to speak up and advocate for my son… Noone else was going to.

(Interview 16, parent)

In one research site, a programme promoting the involvement of families in patient care and acknowledging and incorporating parent perceptions into care planning was being rolled out. This was on the back of adverse events reported on the site, and similar efforts were not observed elsewhere.

They're not the enemy, y'know?

(Interview 11, Medicines Safety Officer)

### 3.2 Agency and Autonomy

Parents and carers functioned autonomously and maintained agency over the care of their child while they were at home, but this autonomy was compromised on admission to hospital; not just with regard to medicines, but to many aspects of care. There were suggestions by the participants that their autonomy was not respected by the organisation. Instead, there was an expectation that parents and carers would assume a passive observer role in their child's care during a hospital episode.

They take the Mum off you when you come in… expect you to just sit back and let them do everything their way…

(Interview 17, Parent)

In spite of this, it was observed that parents would often be involved in medication processes with their children. This was particularly evident in those families with experience of hospitalisation. Primarily, this was to support nursing staff in managing their workloads. Nursing staff were also clear that, without parental support, many medicines would just not be given.

They're the ones that know their child best, they know what they're doing, and it means one less thing for me to do on my shift…

(Fieldnote discussion, Nurse, CH2)

Professionals would describe the care they were providing as being ‘patient‐centred’, but the experience of families was that this commitment was often superficial and inconsistently applied. Several families described their experiences with medication administration when they were initially admitted.

Every time we come in here it all falls apart. They prescribe his meds at random times, and we have a really strict routine at home. I know that schedules and routines are different here, but if he doesn't get his nitrazepam on time he gets dystonic and it just makes everything worse…

(Fieldnote discussion, Parent, CH1)

He has his Movicol with his tea at 5 or 6 pm, with a glass of juice… but when he was in this hospital they gave it to him at 8 or 9 pm with water and… it was just really disorienting for him…

(Interview 16, Parent)

However, there was a suggestion that the system was not calibrated for such patient‐centredness.

We try to be patient centred, and focus on family routines, but the problem is it's just too ad hoc. We're giving medicines at all hours of the day or night… there's another ward that has a traditional medicines round with a trolley… it all feels a bit more controlled.

(Fieldnote discussion, Senior Nurse, CH2)

On admission to the hospital, medication orders were usually made at the convenience of the prescriber (often a medical professional) and were documented contemporaneously – that is, the orders commenced at the point of prescribing. It was unusual for medical staff to account for home routine at this point in the patient's stay. When documenting medication histories for patients on admission, it was common for medical staff to deprioritise the detail because it was expected that the pharmacy service will intervene to make amendments later.

To be honest on a nightshift, we're just firefighting… we don't have time for the detail and the pharmacy will just review it and pick anything up in the morning. I know it's not good practice but…[shrugs].

(Fieldnote discussion, Junior doctor, CH1)

It was also noted that where electronic prescribing was the norm, times of medication administration often defaulted to a computer‐calculated time – be that based on the time of entry, or on pre‐defined times established in the system.

Like, I know they're busy, right? But the protocols just default to a standard set of times, and they don't always remember that so they just assume that things are prescribed properly when the timings are all off.

(Interview 8, Pharmacist)

Yet there was a desire for clinicians to take more notice of family routines, and incorporate them into clinical routines, but there was a need for some incentive to do that.

…like last month I completed an excellence form for a junior [doctor] who did a really good drug history and prescribed everything at the times that the child would have them at home…

(Interview 7, Consultant)

While parents were often co‐opted to administer medicines by clinical staff, this was in contradiction to organisational expectations where parent administration was something that needed to be controlled and managed. Two sites had formal policies for parent administration of medicines, and parent medication errors were noted to be a problem in both sites, however there was a suggestion that this was because the expectations of clinicians on parents were not made clear.

The problem is we don't ask the right questions. You get these incident reports where they blame the parents for giving the medicine and you do some factfinding… and it's great that they've got that relationship and rapport but ultimately… you're the registrant. You're responsible.

(Interview 14, Nurse)

This absence of instruction led parents to administer medicines to their children whether they were prescribed or not. There was no malice observed in this activity – it was just parents continuing to care for their children in a new place. A child's journey through the healthcare system was long and characterised with several stops along the way – the emergency department, the assessment unit, the ward. We observed parents continuing to give medicines throughout this journey until they were explicitly advised to stop. Conversely, there was an assumption on the part of clinical staff that medicines would not be administered if they were not prescribed.

Oh yeah, we got here at 6am and I've given her a change and a feed and her vitamins and folic acid… of course I'll tell the doctor when I see one… what time is it now? 1 pm?

(Fieldnote discussion, Parent, CH1)

Methylphenidate was marked as “omitted/drug unavailable” over a weekend and nursing staff asked the pharmacist to requisition some on Monday morning. After identifying an issue with the formulation prescribed, the pharmacist spoke with the patient's carer. It was identified that the carer had the medicine in their handbag and had administered it every morning over the weekend. “Well, no one asked if I had any, or if he was still getting it. I didn't think there was a problem.”

(Fieldnote observation, CH2)

This suggests that the emergency department, the assessment unit and the ward may function as isolated systems with their own objectives. However, parents viewed the hospital as a single system and would just carry on their usual care until advised otherwise or, as was observed above, as a way of exerting control over their child's care in what they considered a chaotic environment.

Oh I do all her own medication, and I keep it all in here as well. If I leave it to the nursing staff everything will be late – 60% within an hour, but the rest… oh my, [we once had] something that was missed and it was found in a syringe under a pillow about 4 h later…

(Fieldnote discussion, Parent, CH2)

This parental autonomy, while being useful in maintaining the care of children in the hospital was also a source of conflict as parental experience would sometimes challenge medical and nursing preconceptions. During one observation, a family was struggling to understand that viral coughs and wheezes may last for 6 or 8 weeks after the initial acute illness. The parents requested multiple interventions including nebulisers, inhalers and cough medicines. This was discussed one evening during a medical handover.

I've explained to Mum, but she's just not listening… and to be honest it's creating some issues. Not ours, but Mum's. She's an Intensive Care nurse and has some views… they're not the right views, but she's quite insistent.

(Fieldnote observation, Handover round, GH1)

However, parents themselves related stories about how they needed to ‘find their voice’ when advocating for their children. They had to learn about the treatment of their children so that they could have conversations with their clinicians.

[After a diagnosis of carbamazepine toxicity] I did my own research and realised that this blood test was done in other countries but not in the UK and certainly not in [my child]. I wasn't impressed.

(Interview 15, Parent)

There appeared to be assumptions made about the level of understanding of parents based on their job roles outside of the care environment. These assumptions may have carried through into their transactions with parents. In the case of the mother complaining about the cough, a junior doctor on the nightshift relayed their discomfort at the parental demands for a cough medicine for the patient.

I'm trying to get through to her that it won't help… but again, she's really fixated on the coughing and then she's working up [her child].

(Fieldnote observation, GH1)

However, it was apparent that the parents had made their own decision about treating the cough.

The ward round commences and the consultant and a registrar go to this patient's room. Child is sat in bed smiling and playing coughing occasionally. Mum and Dad are present. Breakfast is on the table by the bed, alongside a bottle of “Bronchostop” cough medicine in a supermarket carrier bag, with a syringe next to it.

(Fieldnote observation, GH1)

There was further evidence that parents accounts of the status of children were overlooked or considered as less important than the impression of a clinician who has little knowledge of the child. Parents relayed experiences where the documented medication intolerances of their child were ignored by staff because they were too busy to really listen to parents.

…it's just because they're too busy so they just take it. Where for me, always being here, I know because obviously the liquid's got a smell and a flavouring, that's why she can't have it. And then I find…so I smell all the medicine before I give it her because the tablet don't have a smell or a flavour.

(Interview 19, Parent)

In some circumstances, parents also felt that they were not listened to as the people who knew their child best.

…I always say the most important thing is for you to listen to me. I'm her mum, I'm with her every day, I can see the changes…

(Interview 19, Parent)

Children also had their own agency, which required parental intervention to manage. In some situations, medicines were offered that were intolerable for children to take, yet in spite of this awareness among healthcare professionals these clinical choices were consistently applied, and it fell to the parent to advocate for their child and ask for something more palatable.

…we use clarithromycin a lot and most of the kids just spit it out. It's grim, it's gritty, it doesn't taste very nice…

(Fieldnote, Conversation with a pharmacist, CH1)

A child is in bed watching TV while the doctor talks to his mother. The doctor is explaining about the choice of antibiotics [clarithromycin is prescribed]. Mum laughs. “Have you ever tried it? It tastes like dog poo.” She makes a retching sound. “In the bin…”.

(Fieldnote, Observation, CH1)

Parental autonomy in the paediatric context can be viewed as a continuum. Some actions are more visible (e.g., when advocating for their children with clinicians) while other acts of autonomy are more subtle; the parents purchasing cough medicine for their child in spite of medical reassurance, and the parent who continues to administer their child's medicines because they haven't been told to stop. This also calls into question the nature of ‘patient centred care’ in a space where all patients have an advocate. These advocates (the parents) have an important role to play in patient safety because they are able to, and do, intervene in order to keep their children safe. However, their uncertain place in this system leads to situations where parents aren't listened to because they are not considered to be part of the system. This results in adverse drug events, even where parents identify that something is wrong.

We had an incident where a parent questioned something. The nurse had it double checked, came back, gave it and it still ended up being wrong.

(Interview 10, Pharmacist)

### 3.3 Communication

This oversight of parental agency and autonomy was perhaps reinforced in the approach to communication with parents and carers. Families encountered a myriad of staff and teams within the hospital system, providing large amounts of information about their child's care, but many of the processes in place to manage this information were disconnected from wider care processes. Wards and departments within hospitals had localised admissions checklists that had to be completed, whether the child had never been in that hospital before, or they had been discharged recently. Medication histories formed a part of these admissions checklists but were also observed to be taken repeatedly by different people at different times.

It's quite frustrating at times because you get the impression that no one knows what they're doing… I'm asked all these questions and I'm, like, I just told that doctor earlier…

(Interview 17, Parent)

However, there was a suggestion that medication in these checklists and assessments were relatively unimportant in comparison with the other information being collected. In one site, the space for documenting the medications taken by the patient was a different size depending on who was completing the form. For medical staff, this space was a two‐inch square box, while for nurses the space was larger but still less than a quarter of a page. The impact of this difference was stark. A patient was admitted with a chest infection who was being managed with warfarin for a previous condition. This anticoagulant was not picked up during the clerking of the patient because the drug history was written on a sticky note and affixed to the inside of the patient's folder with the expectation that ‘someone else’ would check it in the morning. No drug chart was completed, and the family continued to administer the medicines overnight. When the lead researcher came to meet the family and look at their medication charts they noticed the discrepancy between what was documented and what was happening. This triggered the only safety intervention in this study, with the observer alerting the consultant and the pharmacist to the patient's condition. When the observer got the chance to speak to the child's parent this experience was quite normal for them.

Yeah I prefer it this way. I know his medicine, I know when to do his blood monitoring and I talk to [the hospital] about dose changes and stuff… we're not in hospital very often, but when we are I do it all. I've handed over the spreadsheet and contact details to the medical team and they're happy for me to carry on.

(Fieldnote discussion, Parent, GH1)

Furthermore, it was observed that parental routines were treated with scepticism by clinicians – medical, nursing and pharmacy staff. All sites required validation of medication histories against multiple sources in the form of clinic letters or printed labels on medication packages, many of which were out of date or inaccurate. A parental history alone was never taken in isolation because practitioners doubted the veracity of these histories. However, families took a different view to these documents. Parental medication histories were based around home routine and habit – mealtimes and school schedules. Additionally, many changes to medication for children were often not documented by clinicians in real time. Medication review was informal and based on individual response at home.

…you know the dose will be tweaked in clinic or the consultant will phone you… and the label from the chemist will never really change…

(Interview 3, Nurse)

Therefore, there is a misalignment between the importance of medication documents for parents and clinicians.

Because of the perceived primacy of documented evidence of medication use, these snippets of parental insight were often ignored or overlooked.

A patient attends for routine blood monitoring of their medication and the doctor taking the blood is pouring over the medical notes. They look up at the healthcare support worker. “Do you know what time the last dose was? It's not in the notes anywhere…?”

Have you asked Mum and Dad? They're giving it…

Yeah but is it written down anywhere? Just to be sure.

(Fieldnote observation, GH1)

## 4 Discussion

This paper is one of the first multicentre ethnographic studies that reports on parent perspectives and experiences of safety, as well as their contributions to the safety of their children in hospitals. Our study sheds new light on the wealth of experience and knowledge regarding care provision parents have which offers an important source of resilience against medication‐associated harm. It also provides important insights on the adaptations that families need to make in order to accommodate hospital medication routines into their home routines. Importantly, there were no substantial differences between the sites within the study as to how these perspectives were manifested, despite there being a range of medicines management technologies deployed (paper and electronic prescribing, traditional storage cabinets and automated dispensing cabinets), thus we can be reasonably assured of some generalisability into other paediatric settings. Other major healthcare ethnographies on system safety have provided only the perspectives of healthcare professionals and organisations in operating theatres, emergency departments, elderly care wards and maternity units [35, 36, 37, 38]. Other ethnographic studies involving patient perspectives on safety have utilised patients and families as analysts in end of life care and infection control as a way of providing additional insight into safety processes in acute wards [39]. We have also demonstrated how health systems subsume and ignore family knowledge and/or routines on admission to hospital which creates confusion and anxiety for parents. Perhaps most importantly, this paper has also identified that parents are systematically excluded from care processes by healthcare systems and expected to be passive observers of their child's care. However, because of systemic weakness in the wider system around availability of resources, parents are informally co‐opted into medicines administration activities.

We have demonstrated that there is a great deal of undocumented and unacknowledged work that families undertake to support their loved ones and keep them safe. O'Hara described this role as ‘scaffolding’ around our systems [40], with parents having a place simultaneously outside, inside and across the boundaries of care such that they provide a space in which healthcare‐related problems can be intercepted and mitigated [41]. Parents act as knowledge brokers with medical teams [42]. Of importance is the contextual information that knowledge brokering provides, which in clinical systems is often lacking or limited. It was seen that parents were available and willing to support medical and nursing staff in solving problems associated with their child's medicines. Similarly, where medical and nursing knowledge and experience did not encompass a child's care, there was a tacit acknowledgement that parents can and do fill that gap.

We found that this ‘reaching in’ by parents for their child's care and advocacy on behalf of their child could be interpreted as the parent manifesting their internal locus of control over their child. It was apparent that on admission to hospital, parents were expected to surrender the independent control over their child's care by healthcare staff in order to maintain patient safety, which was mostly done with understanding and grace. However, where care‐related issues began to emerge – for example the omission of medicines or disagreement over how medicines would be given – then parents would begin to manifest their control over their child's care with expectations of independence.

However, this often created conflict, with the parental locus of control treated with suspicion and/or disregarded and therefore subsumed within the clinical locus of control of the wider multi‐professional paediatric teams. We contend that this is a manifestation of epistemic injustice. This is defined as ‘a wrong done to someone in their capacity as a knower’ [43]. In our study, it is manifested as a failure by professionals to believe the patients and parents because of structural prejudices related to the power structures intrinsic in healthcare systems. This could be argued as an example of testimonial injustice and is an emerging area of interest in healthcare research [44, 45]. The needs, practices and opinions of patients and families were rejected or viewed with suspicion by the organisations responsible for providing care because there was a lack of control and assurance over them. However clinical staff would often rely on parents to support them in a tacit acknowledgement of parental expertise. Notwithstanding this informal adaptation, there were occasions where this expertise was rejected at the bedside because of how clinical staff assessed what was ‘normal’ work [46]. This is now more important than ever in the United Kingdom with well‐documented episodes of parental dismissal leading to catastrophic outcomes and changes in UK health law [47, 48]. We contend that epistemic injustice is pervasive in a system that is not equipped to deal with patients who have competent and powerful advocates. The involvement and promotion of patients and families in maintaining paediatric patient safety can no longer be ignored [49]. We recommend future qualitative exploration of the impact of conflicting locus‐of‐control between paediatric healthcare teams and families, and how these impact the construction and maintenance of patient safety.

What our study has helped to illuminate is that parents adapt their approach to suit home routines and the individual needs of their children, and how these are viewed as ‘wrong’ by medical and nursing staff on admission to the hospital. We are only aware of a single study that explores the ‘work as done’ of parents with CMC who require medication at home. Abebe used Work Domain Analysis (WDA) to understand how parents adapted within the constraints of medication management for ambulatory CMC and identified managing medication supply and administration as the main areas for adaptation [50]. Critically, this article identified that parents were capable of managing their children's medicines but that this was not acknowledged or supported by healthcare professionals. We follow on from this study with the clear suggestion that these adaptations are essential but are not necessarily continued when children are in hospital, which creates anxiety and confusion for families.

We have also confirmed that parents administering medicines to children in hospital is routine, despite organisational denial that this occurs. This was first implied over ten years ago when Alsulami et al. studied the adherence of nursing staff to second‐checking policies and identified that unsupervised parent administration of medicines was the single most common medication ‘error’ to be observed [51]. While we observed nursing staff offering a number of justifications for allowing that, we posit that this is a natural situational adaptation by nursing staff to account for a relative lack of nursing resources and has become a natural ‘first option’. It should not be categorised as a medication error and offers potential opportunities for medicine safety and improved family satisfaction of healthcare services.

This falling back onto parents of medication administration could be viewed as an exemplar of the tension between organisational expectations and service needs on the ground. Another example of this is the organisational concern about parental knowledge, competency and ability, manifested through the rigorous validation of parent history against prior clinical documentation and medical records. It is well understood that medication records for children and young people are often inaccurate or incomplete; however, it has been demonstrated in several well‐designed studies that parental medication histories are often more accurate than those documented in the medical notes [52, 53, 54]. Terry et al. identified that with respect to parental autonomy and agency the medication history reported by a parent is likely to be the best possible medication history as it represents a real‐world account of child medication administration [55]. Our study has demonstrated how in spite of this knowledge, parental medication histories are treated with suspicion until confirmed against a false dataset. Given the large expenditure of resources and prioritisation of medicines reconciliation as a medicines safety intervention, there is a need for further study on the involvement of parents in maintaining patient records, and the potential efficiencies therein.

Overall, we believe that this study suggests that the involvement of parents and carers in medication administration may support and maintain medicine safety for children. Self‐Administration of Medicines schemes are ‘standard of care’ for adult patients in hospital, but the benefits of these schemes on outcomes for medication safety (i.e. reduced medication errors) are unclear [56]. There are no robust studies pertaining to parent administration of medicines demonstrating a clear advantage for parent administration, however a German pilot study of the implementation of an educational intervention to support parent administration of enteral medicines demonstrated a significant reduction in the incidence of observed medication errors among parents [57]. However, this study was underpowered (only 30 parents were observed) and a similar reduction in errors was observed in the nursing cohort. A more recent small‐scale study identified other benefits of parent self‐medication in a cohort of cystic fibrosis patients in a British tertiary facility, with improved discharge efficiency, family‐clinician communication, and reduced costs [58]. We therefore recommend that there should be further research to co‐develop and test an intervention to support parental self‐administration of medicines in hospitals as this may offer benefits in reducing the risks of epistemic injustice and promote parent confidence in healthcare systems. However, because of the lack of prior empirical study of this as a concept we also recommend that any study consider the benefits and opportunities, and barriers and risks associated with parent administration of medicines in hospital.

### 4.1 Strengths and Limitations

The strength of this study lies within its use of focused multi‐site ethnographic observations supported by semi‐structured interviews which enabled both breadth and depth of data collection and the incorporation of multiple perspectives across staff and parents. However, the study utilised only a single observer which may have limited the acquisition of data relating to specific aspects of the field. This observer is a man in his mid‐forties who does not have children of his own, so a degree of objectivity in the research may have come from this. Further mitigations to this limitation included the involvement of a wide range of methodological experts in the analysis and the incorporation of the parent and family voice in the analysis and interpretation of the data in this study providing some assurance of the credibility of the data and its meaning.

While there were many anecdotes of adverse drug events it was not possible for this study to observe the emergence of safety events to their logical conclusion. In one event where there was the sign of emergence, the potential outcomes for the patient in those circumstances required observer intervention to prevent any harm. This reflects the difficult position of observational safety research – serious events and patient harm especially in children appear to be relatively rare, therefore, to robustly study adverse drug events, observers must be ‘in the right place, at the right time’. Conversely, it would be ethically and professionally difficult for observers to justify allowing the event to play out.

## 5 Conclusions

Medication safety for children in hospitals is a social phenomenon constructed through the interaction of parents and healthcare professionals. Parents and families, however, are operationally and organisationally excluded from the system and are treated with suspicion, partly because their home routines do not conform to biomedical norms. These routines are often disregarded on admission and replaced by hospital systems and norms, which leads to parent anxiety and confusion. This is exacerbated by a lack of clear expectations of parents. Parents and carers usually want to participate in the care of their children and do so when it suits healthcare staff to meet their objectives, but where objectives or opinions differ then parent perspectives are overridden or ignored. Parents offer resilience and potential safety benefits for medicines safety for hospitalised children and young people and this should be utilised more effectively in practice.

## Author Contributions

**Adam Sutherland:** conceptualisation, investigation, funding acquisition, writing–original draft, methodology, formal analysis, project administration, data curation. **Denham L. Phipps:** investigation, methodology, validation, writing–review and editing, supervision. **Stephen Tomlin:** supervision, formal analysis, writing–review and editing. **Suzanne Grant:** methodology, writing–review and editing, formal analysis. **Joanne Hughes:** writing–review and editing, formal analysis, validation. **Joanna Chambers:** writing–review and editing, formal analysis, validation. **Susan Kafka:** validation, writing–review and editing, formal analysis. **Heidi Ridgewell:** writing–review and editing, validation, formal analysis. **Darren M. Ashcroft:** funding acquisition, methodology, writing–review and editing, formal analysis, project administration, supervision.

## Conflicts of Interest

The authors declare no conflicts of interest.

## Data Availability

Anonymised data is freely available through FigShare at the following DOI: 10.48420/24925329.v1.
